# Evaluation of SAMP8 Mice as a Model for Sleep-Wake and Rhythm Disturbances Associated with Alzheimer’s Disease: Impact of Treatment with the Dual Orexin (Hypocretin) Receptor Antagonist Lemborexant

**DOI:** 10.3233/JAD-201054

**Published:** 2021-06-01

**Authors:** Carsten T. Beuckmann, Hiroyuki Suzuki, Erik S. Musiek, Takashi Ueno, Toshitaka Sato, Masahiro Bando, Yoshihide Osada, Margaret Moline

**Affiliations:** a Eisai Co., Ltd., Tsukuba, Ibaraki, Japan; b Department of Neurology, Washington University School of Medicine, St. Louis, MO, USA; c Eisai Inc., Woodcliff Lake, NJ, USA

**Keywords:** Alzheimer’s disease, animal models, dual orexin receptor antagonist, E2006, *in vivo*, irregular sleep-wake rhythm disorder, lemborexant, mouse, orexin, running wheel, sleep

## Abstract

**Background:**

Many patients with Alzheimer’s disease (AD) display circadian rhythm and sleep-wake disturbances. However, few mouse AD models exhibit these disturbances. Lemborexant, a dual orexin receptor antagonist, is under development for treating circadian rhythm disorders in dementia.

**Objective:**

Evaluation of senescence-accelerated mouse prone-8 (SAMP8) mice as a model for sleep-wake and rhythm disturbances in AD and the effect of lemborexant by assessing sleep-wake/diurnal rhythm behavior.

**Methods:**

SAMP8 and control senescence-accelerated mouse resistant-1 (SAMR1) mice received vehicle or lemborexant at light onset; plasma lemborexant and diurnal cerebrospinal fluid (CSF) orexin concentrations were assessed. Sleep-wake behavior and running wheel activity were evaluated.

**Results:**

Plasma lemborexant concentrations were similar between strains. The peak/nadir timing of CSF orexin concentrations were approximately opposite between strains. During lights-on, SAMP8 mice showed less non-rapid eye movement (non-REM) and REM sleep than SAMR1 mice. Lemborexant treatment normalized wakefulness/non-REM sleep in SAMP8 mice. During lights-off, lemborexant-treated SAMR1 mice showed increased non-REM sleep; lemborexant-treated SAMP8 mice displayed increased wakefulness. SAMP8 mice showed differences in electroencephalogram architecture versus SAMR1 mice. SAMP8 mice exhibited more running wheel activity during lights-on. Lemborexant treatment reduced activity during lights-on and increased activity in the latter half of lights-off, demonstrating a corrective effect on overall diurnal rhythm. Lemborexant delayed the acrophase of activity in both strains by approximately 1 hour.

**Conclusion:**

SAMP8 mice display several aspects of sleep-wake and rhythm disturbances in AD, notably mistimed activity. These findings provide some preclinical rationale for evaluating lemborexant in patients with AD who experience sleep-wake and rhythm disturbances.

## INTRODUCTION

Approximately 45%of patients with Alzheimer’s disease (AD) display circadian rhythm and sleep-wake disturbances [1], which may precede overt cognitive impairments [2, 3]. When these disturbances occur frequently, they can be collectively referred to as irregular sleep-wake rhythm disorder (ISWRD; ICD-10-CM code G47.23). This circadian rhythm sleep disorder is also identified in the *Diagnostic and Statistical Manual of Mental Disorders, 5th Edition* [4] and the *International Classification of Sleep Disorders, 3rd Edition* [5]. The typical symptoms include fragmented sleep, frequent nighttime awakenings, unintended bouts of sleep during the day, and excessive daytime sleepiness [6–8]. Sleep-related aspects of ISWRD have been observed in up to 66%of patients with AD [9]. Alterations in sleep architecture may also occur in AD, specifically reduced rapid eye movement (REM) and slow wave sleep, and increased REM sleep latency [6, 10, 11]. Increased nighttime wakefulness and activity are particularly difficult symptoms for caregivers, and sleep issues often ultimately contribute to the institutionalization of patients with AD [7, 12]. Treatments used for sleep disturbances in AD are often problematic (e.g., benzodiazepines and non-benzodiazepines have been found to increase the risk of fractures in patients with dementia) [13], and are not recommended by the American Geriatrics Society (“Beers list”) for use in older adults for this and other reasons [14].

Senescence-accelerated mouse prone-8 (SAMP8) mice, created by Kyoto University (Japan) by selective breeding [15, 16], display AD-like learning and memory deficits compared with their control, senescent-accelerated mouse resistant-1 (SAMR1), counterparts [17]. SAMP8 mice also exhibit other AD-like characteristics, including increased locomotion behavior at the onset of the rest (lights-on) period, which is potentially analogous to “sundowning” in patients with AD [18, 19]. These mice also show various pathological changes, such as increased amyloid-β protein precursor, increased amyloid-β (Aβ) protein, amyloid-like plaque deposits, hyperphosphorylation of tau protein, decreased dendritic spine density, and decreased choline acetyl transferase activity [16, 17, 19]. Pathophysiologic and cognition/memory disturbances are the typical areas of focus in mouse models of AD. In contrast, models focused on the sleep-wake and rhythm phenotypes of AD are scarce. Indeed, few mouse AD models exhibit robust and consistent sleep and circadian deficits [20]. We therefore wanted to investigate whether SAMP8 mice exhibit some of the disturbed sleep-wake and rhythm aspects of AD.

Lemborexant (E2006) is a highly specific dual orexin (hypocretin) receptor antagonist (DORA; K_i_ =4.8 nmol/L for human orexin-1 receptor; K_i_ = 0.61nmol/L for human orexin-2 receptor [21]) that is approved for the treatment of insomnia and is currently in clinical development for the treatment of ISWRD. Preclinical studies in mice and rats demonstrated that lemborexant promoted both REM and non-REM sleep, and that this was mediated via the orexin signaling pathway, based on the observed absence of sleep promotion in orexin neuron- and peptide-deficient mice [22, 23]. Phase II and III clinical studies have demonstrated the efficacy and tolerability of lemborexant 5 mg and 10 mg in patients with insomnia [24–26].

The objectives of the studies reported herein were to characterize sleep-wake and rhythm behavior of SAMP8 mice and to investigate the effect of lemborexant. For this, we assessed the plasma concentration profile and pharmacokinetics (PK) of lemborexant, orexin-A (OXA) concentration profile in the cerebrospinal fluid (CSF), sleep-wake behavior, and diurnal activity rhythm by wheel running measurements. Given the finding of decreased A_β_ plaque formation in another AD mouse model following dosing with almorexant [27], another DORA, testing lemborexant in this animal model SAMP8 was predicted to provide important information about the potential for DORAs to ameliorate some of the symptoms of AD.

## MATERIALS AND METHODS

### Animals

Animal care and experimental procedures were performed in an animal facility accredited by the health science center for accreditation of laboratory animal care and use of the Japan health sciences foundation. Experimental protocols were approved by the Institutional Animal Care and Use Committee of Eisai Co., Ltd., Tsukuba Research Laboratories. SAMP8 and SAMR1 mice were supplied by Japan SLC, Inc. (Shizuoka, Japan) and maintained under a 12-h light-dark cycle with food and water available *ad libitum*.

### PK of lemborexant in SAMP8 and SAMR1 mice

Male SAMP8 and SAMR1 mice (age, 22 weeks; body weight, 27.8–43.7 g; *n* = 3 per strain per time point) were dosed orally with lemborexant 30 mg/kg at the onset of light (Zeitgeber time [ZT] 0:00–0:30). Blood samples were collected from the abdominal aorta or vein (under isoflurane anesthesia) using heparinized syringes at 0.25, 0.5, 1, 2, 3, 6, 9, 12, 15, 18, 21, and 24-h (3 mice per strain per time point). Samples were centrifuged, and plasma aliquots used for the assessment of lemborexant PK parameters.

Plasma concentrations of lemborexant were measured using liquid chromatography-tandem mass spectrometry (LC-MS/MS). Individual mouse plasma samples were precipitated with acetonitrile containing deuterium-labeled lemborexant as an internal standard. After mixing and centrifugation, the resulting supernatant was filtered and subjected to LC-MS/MS. Detection was accomplished by multiple reaction monitoring in positive ionization mode. The ratio of the peak area responses relative to the internal standard were used to construct a standard curve using linear least squares regression with a 1/x^2^ weighting. Plasma concentrations are reported as mean±standard deviation. PK parameters, maximum drug concentration (C_max_), area under the concentration-time curve (AUC), and time to reach maximum (peak) concentration following drug administration were determined based on the mean plasma concentration.

### Evaluation of diurnal orexin concentrations and effect of lemborexant on diurnal OXA concentrations in SAMP8 and SAMR1 mice

Male SAMP8 and SAMR1 mice (age, 22 weeks; body weight, 27.8–43.7 g) were dosed orally with vehicle (0.5%[w/v] methylcellulose, 10 mL/kg) or lemborexant 30 mg/kg at the onset of light (ZT 0:00–0:30). CSF was collected from the cisterna magna every 3-h at ZT 3, 6, 9, 12, 15, 18, 21, and 24 (*n* = 3 per strain and treatment per each time point). Anesthetized mice (midazolam, medetomidine hydrochloride, butorphanol tartrate) were fixed in a stereotaxic frame, the neck skin was incised, and the neck muscles were pulled to the sides until the base of the skull was exposed. A glass capillary was then inserted into the cisterna magna, and 9–17μL of CSF was collected from each animal and deposited into a protein low-binding tube. The capillary was then washed three times with three volumes of acetonitrile, and wash fluid was added to the tube containing the CSF.

OXA concentrations were measured by enzyme-linked immunosorbent assay kit (FUJIFILM Wako Pure Chemical Corporation, Osaka, Japan). After addition of dimethyl sulfoxide, CSF samples were incubated at 37°C to evaporate acetonitrile and then reconstituted with buffer supplied with the kit.

### Effect of lemborexant on vigilance states in SAMP8 and SAMR1 mice

Male SAMR1 mice (age, 18–19 weeks; body weight, 33.0–39.5 g; *n* = 8) and SAMP8 mice (age, 18–19 weeks; body weight, 27.4–35.0 g; *n* = 8) were implanted with transcranial epidural electroencephalography (EEG) and intranuchal electromyography (EMG) electrodes as previously detailed [22, 28, 29]. Briefly, four holes were drilled into the skull, and four contacts of a six-contact board mount socket were inserted into the dura mater. The remaining two contacts were placed intranuchally into pockets formed by blunt dissection of neck muscle left and right of midline. EEG signals were read from one hemisphere of the brain only, between the rostral and caudal electrodes. Signals were collected via a connector attached to the board mount socket, which was connected to an amplifier via a rotating swivel, thereby allowing mice to move freely during recovery and experiments. After 2–3 weeks of recovery, a randomized crossover design was implemented, where mice (*n* = 8 per strain) were dosed orally with vehicle (0.5%[v/v] methylcellulose, 10 mL/kg), lemborexant 3 mg/kg, and lemborexant 30 mg/kg at the onset of light (ZT 0:00–0:30) over 3 consecutive days. EEG/EMG signals were continuously recorded up to 24-h after the final dose.

EEG/EMG signals were divided into 10-s epochs, and analyzed as previously described [29], per standard rodent sleep criteria [30], using SleepSign software (v3, Kissei Comtec, Nagano, Japan). Automatically analyzed data were visually verified by a trained observer. Sleep latency was defined as time from dosing to occurrence of first 270 s of sleep (with up to two epochs of intermittent wakefulness permitted). REM sleep latency was defined as time from dosing to first occurrence of a REM sleep epoch. Bouts of wakefulness, non-REM sleep, and REM sleep were defined as being in that respective vigilance state without interruption. Minimum bout duration was one epoch (10 s), with no limit to maximum duration. Average bout duration and bout counts for wakefulness, non-REM sleep, and REM sleep were calculated using SleepSign analysis software. Total bout counts were calculated as the sum of the respective wakefulness, non-REM sleep, and REM sleep bout counts. EEG power spectra were calculated in 1-Hz bins from 0–30 Hz for the lights-on and lights-off periods and divided into the respective vigilance states, either normalized to maximum signal intensity of peak bin for every strain/treatment cohort (for peak frequency comparison), or raw signal intensities used (for power comparison), and averaged (SAMP8 *n* = 8, SAMR1 *n* = 7 [one SAMR1 animal was removed from analysis due to EEG artifacts]). EEG bands were defined as delta (1–4 Hz), theta (4–8 Hz), alpha1 (8–11 Hz), alpha2 (11–13 Hz), beta1 (13–22  Hz), and beta2 (22–30 Hz) [31]. The diurnal ratio of wakefulness was calculated as wakefulness time ratio of total wakefulness time over the diurnal day in percent during the light-off period.

### Effect of lemborexant on diurnal rhythm of voluntary running wheel activity in SAMP8 and SAMR1 mice

Male SAMP8 and SAMR1 mice (age, 20–21 weeks; body weight, 28.3–44.4 g; *n* = 23–24 per strain) were individually housed in plastic cages with running wheels (RWC-15, Melquest, Toyama, Japan). Baseline (pretreatment) assessments were performed for 10 days without dosing, of which days 3–10 were used for analysis. Mice were then dosed orally with vehicle (0.5%[v/v] methylcellulose, 10 mL/kg) or with lemborexant 30 mg/kg at the onset of light (9:00 AM [ZT 0:00–0:30]) each day and assessed for a further 10 days. Posttreatment assessments (no dosing) were then performed for 10 days. All recordings were performed under 12-h light:12-h dark conditions.

Running wheel activity in 1-min bins was monitored using an Actmaster 4 (Melquest, Toyama, Japan). Scores for individual 24-h profiles were averaged for each group of mice. Activity data were analyzed using ClockLab software (version 6.0.54, Actimetrics, Wilmette, IL, USA). Parameters assessed included acrophase, period, amplitude, and variability of diurnal activity rhythm.

Data from baseline days 1 and 2 were discarded due to acclimation effects and variability of activity start/end times. Acrophase was calculated as the peak of a sine curve fit to each day of activity data for individual mice or normalized average data for each cohort. Intradaily variability (IV), interdaily stability (IS), and onset of activity were automatically determined using the ClockLab algorithm for each individual animal.

### Statistical analysis

Statistical analyses were performed using: 1) linear mixed-model analysis (with treatment and time as fixed effects and animal as a random effect) for log-transformed 3-h OXA data and two-way analysis of variance followed by Fisher’s least significance difference test for log-transformed 12-h OXA data; 2) linear mixed-model analysis (with treatment and strain as fixed effects and animal as a random effect, assuming variance component as the variance structure) for vigilance state time data, log-transformed pretreatment total activity running wheel count data, and running wheel activity data (acrophase, IV, IS); 3) linear mixed-model analysis (with treatment and strain as fixed effects and animal and time-bin as random effects, assuming variance component as the covariance structure) for log-transformed activity counts per 6-h bin data; 4) linear mixed-model analysis (with treatment and strain as fixed effects and animal and spectra band as random effects, assuming variance component as the covariance structure) for EEG band power spectra analysis data (absolute and normalized); and 5) unpaired *t*-test for running wheel activity IV, IS, and acrophase data. The denominator degree of freedom for mixed models was calculated by Kenward–Roger’s method. For crossover designs data, models included experimental period as a covariate. Multiplicity adjustment for more than two doses was conducted by Dunnett’s or Holm’s method. For all analyses, *p* < 0.05 (two sided) was taken to indicate statistical significance. Analyses were performed using SAS software (version≥9.3, SAS Institute Inc., Cary, NC, USA).

## RESULTS

### PK of lemborexant in SAMP8 and SAMR1 mice

**Fig. 1:**
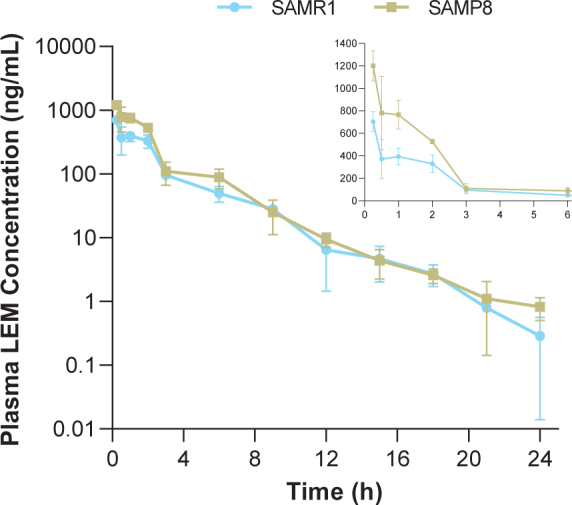
Time course of plasma lemborexant concentrations in senescence-accelerated mouse prone-8 (SAMP8) and senescence-accelerated mouse resistant-1 (SAMR1) mice (*n* = 3 per strain) after a single oral dose of lemborexant 30 mg/kg. Main figure shows plasma lemborexant (LEM) concentrations from 0 to 24-h (log scale). Inset figure shows plasma LEM concentrations from 0 to 6-h.

In both strains, time to reach maximum (peak) concentration following drug administration after a single oral dose of lemborexant 30 mg/kg was 0.25-h at the first sample time point (Table 1, Fig. 1). Plasma concentrations in SAMP8 mice were approximately two-fold higher than those in SAMR1 mice shortly after dosing (Fig. 1 inset). Thereafter, plasma concentrations were similar between strains. C_max_ and AUC from time 0–24-h in SAMP8 mice were 1.7- and 1.6-fold higher, respectively, than in SAMR1 mice (Table 1).

**Table 1:** Lemborexant pharmacokinetic parameters in SAMP8 and SAMR1 mice after a single oral dose of lemborexant 30 mg/kg

Parameter	SAMR1 mice	SAMP8 mice
C_max_ (ng/mL)	704	1,200
t_max_ (h)	0.25	0.25
AUC_(0–24-h)_ (ng h/mL)	1,410	2,310

AUC_(0–24-h)_, area under the concentration-time curve from time 0–24-h; C_max_, maximum drug concentration; SAMP8, senescence-accelerated mouse prone-8; SAMR1, senescence-accelerated mouse resistant-1; t_max_, time to reach maximum (peak) concentration following drug administration.

### Diurnal orexin concentrations and effect of lemborexant on diurnal orexin concentrations in SAMP8 and SAMR1 mice

**Fig. 2:**
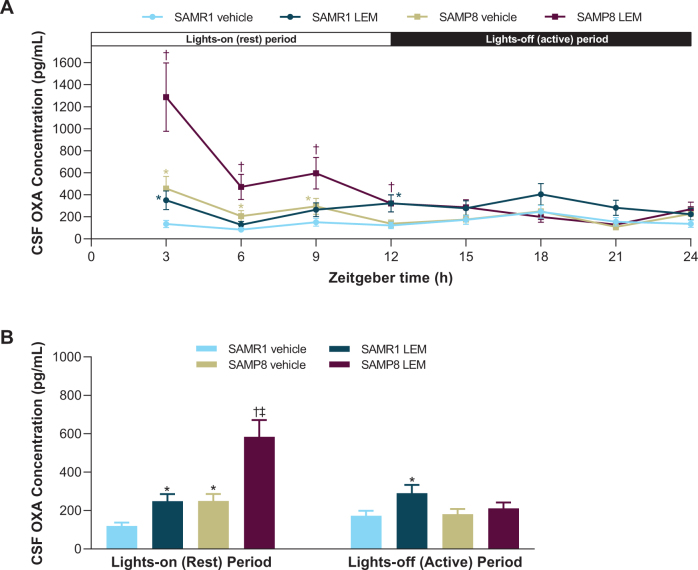
A) Effect of lemborexant (LEM) on cerebrospinal fluid (CSF) orexin-A (OXA) concentrations over 24-h and B) averaged CSF OXA concentrations during the lights-on and lights-off periods in senescence-accelerated mouse prone-8 (SAMP8) and senescence—accelerated mouse resistant-1 (SAMR1) mice. Mice (*n* = 3 per strain, per treatment) received single oral doses of vehicle or LEM 30 mg/kg at Zeitgeber time 0:00–0:30. Data are mean±standard error of the mean. ^*^p < 0.05 versus SAMR1 vehicle. ^†^*p* < 0.05 versus SAMP8 vehicle. ^‡^*p* < 0.05 versus SAMP8 LEM lights-off period. Statistical analyses were performed using: A) linear mixed-model analysis (with treatment and time as fixed effects and animal as a random effect) for log-transformed 3-h OXA data, B) two-way analysis of variance (strain and treatment as main effect), followed by Fisher’s least significance difference test for log-transformed 12-h OXA data in each period.

OXA concentrations in vehicle-treated SAMR1 mice were lowest in the middle of the lights-on period (ZT 6) and peaked in the middle of the lights-off period (ZT 18; Fig. 2A). In contrast, OXA concentrations in vehicle-treated SAMP8 mice were lowest in the lights-off period (ZT 21) and peaked in the lights-on period (ZT 3; Fig. 2A). When averaged over the respective 12-h light-dark periods, vehicle-treated SAMP8 mice had approximately twice as much OXA in the CSF than vehicle-treated SAMR1 mice during the lights-on period (*p* < 0.05), while there was no significant difference during the lights-off period (Fig. 2B). After treatment with lemborexant 30 mg/kg, both strains displayed an immediate and significant increase in CSF OXA concentration (Fig. 2).

### Effect of lemborexant on vigilance states in SAMP8 and SAMR1 mice

**Fig. 3:**
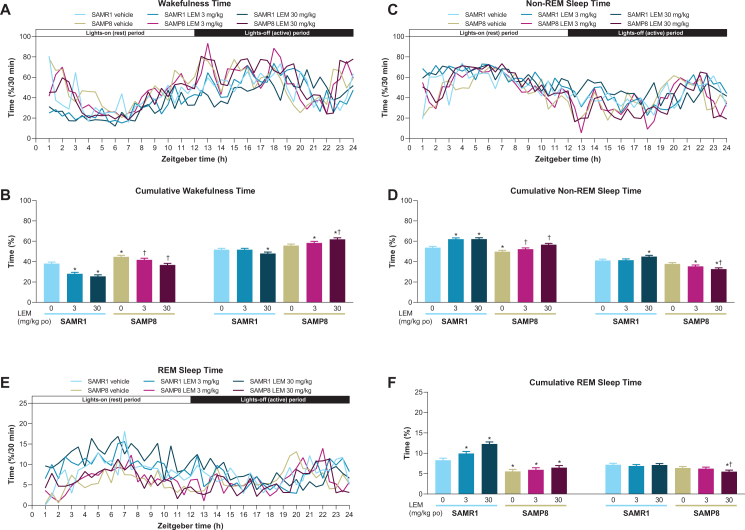
A, C, E) Effect of lemborexant (LEM) on vigilance states over 24-h and B, D, F) cumulative vigilance states in senescence-accelerated mouse prone-8 (SAMP8) and senescence-accelerated mouse resistant-1 (SAMR1 mice). In a randomized crossover design, mice (*n* = 8 per strain, per treatment) received single oral (po) doses of vehicle, LEM 3 mg/kg, and LEM 30 mg/kg at Zeitgeber time 0:00–0:30. Data are mean±standard error of the mean. ^*^p < 0.05 versus SAMR1 vehicle. ^†^*p* < 0.05 versus SAMP8 vehicle. REM, rapid eye movement. Statistical analyses were performed using: B, D, F) linear mixed-model analysis (with treatment and strain as fixed effects and animal as a random effect), followed by Fisher’s least significance difference test (SAMR1-vehicle versus SAMP8-vehicle) or Dunnett type multiple comparison test (versus vehicle).

Vehicle-treated SAMP8 mice showed more wakefulness during the first half of both periods compared with vehicle-treated SAMR1 mice (Fig. 3A), and more total wakefulness time over the entire diurnal day (Supplementary Figure 1). Lemborexant dose-dependently decreased wake time during the lights-on period in both strains (Figs. 3A, B). Findings during the lights-off period differed by strain, with lemborexant 30 mg/kg decreasing wakefulness in SAMR1 mice, but increasing wakefulness in SAMP8 mice (Figs. 3A, B). Due to this strain-specific effect in the lights-off period, lemborexant significantly reduced total wakefulness time over 23.5-h in SAMR1 mice but did not change total wakefulness time in SAMP8 mice (Supplementary Figure 1). When calculating the diurnal wakefulness ratio as wake time percent during the active period (which is comparable with “relative amplitude” measured in AD patients), SAMP8 mice did not show a significant difference from SAMR1 mice, whereas lemborexant significantly increased the diurnal wake ratio in both strains (Supplementary Figure 2). Therefore, for SAMP8 mice, lemborexant did not change total wakefulness time over the entire diurnal cycle, but shifted time spent in wakefulness from the lights-on (resting) period to the lights-off (active) period.

In keeping with the wakefulness findings, SAMP8 exhibited less non-REM and REM sleep at the beginning of both periods compared with SAMR1 mice (Figs. 3C-F). The between-strain difference was more apparent for REM than non-REM sleep. Also consistent with the wakefulness findings, lemborexant dose-dependently increased non-REM (Fig. 3D) and REM (Fig. 3F) sleep during the lights-on period in SAMR1 mice and non-REM sleep in SAMP8 mice (Fig. 3D). Findings during the lights-off period again differed by strain, with lemborexant 30 mg/kg increasing non-REM sleep, but not REM sleep in SAMR1 mice, while decreasing both non-REM and REM sleep in SAMP8 mice (Fig. 3C-F).

Lemborexant 30 mg/kg caused sporadic sleep-onset REM (SOREM) events in both strains. Specifically, two of eight SAMR1 mice experienced three events, while three of eight SAMP8 mice experienced five events during the lights-on period. One vehicle-treated SAMP8 mouse had four SOREM events during the lights-off period.

**Fig. 4:**
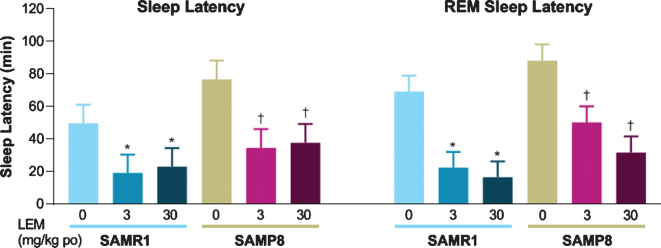
Effect of lemborexant (LEM) on sleep latency and rapid eye movement (REM) sleep latency in senescence-accelerated mouse resistant-1 (SAMR1) and senescence-accelerated mouse prone-8 (SAMP8) mice. In a randomized crossover design, mice (*n* = 8 per strain, per treatment) received single oral (po) doses of vehicle, LEM 3 mg/kg, and LEM 30 mg/kg at Zeitgeber time 0:00–0:30. Data are mean±standard error of the mean. ^*^p < 0.05 versus SAMR1 vehicle. ^†^*p* < 0.05 versus SAMP8 vehicle. Statistical analyses were performed using linear mixed-model analysis (with treatment as fixed effects and animal as a random effect), followed by Dunnett type multiple comparison test in each strain.

Both sleep latency and REM sleep latency after dosing with vehicle were numerically longer in SAMP8 mice compared with SAMR1 mice (Fig. 4). Lemborexant dose-dependently reduced sleep latency and REM sleep latency in both strains.

When analyzing fragmentation of sleep-wake behavior by evaluating vigilance bout counts and duration in the lights-on period, lemborexant increased bout counts (Supplementary Figure 3A), but reduced bout duration only during wakefulness (Supplementary Figure 3B), leading to more overall state transitions. There were no significant indications of heightened fragmentation of sleep-wake behavior in vehicle-treated SAMP8 mice compared with vehicle-treated SAMR1 mice in the lights-off period (Supplementary Figure 3C, D). The observed increase in wakefulness time in SAMP8 mice during the lights-out period (Fig. 3B) seems to be caused by a reduction of bout counts (Supplementary Figure 3C) and an increase in bout duration (Supplementary Figure 3D), i.e., lemborexant consolidated wakefulness in SAMP8 mice.

**Fig. 5:**
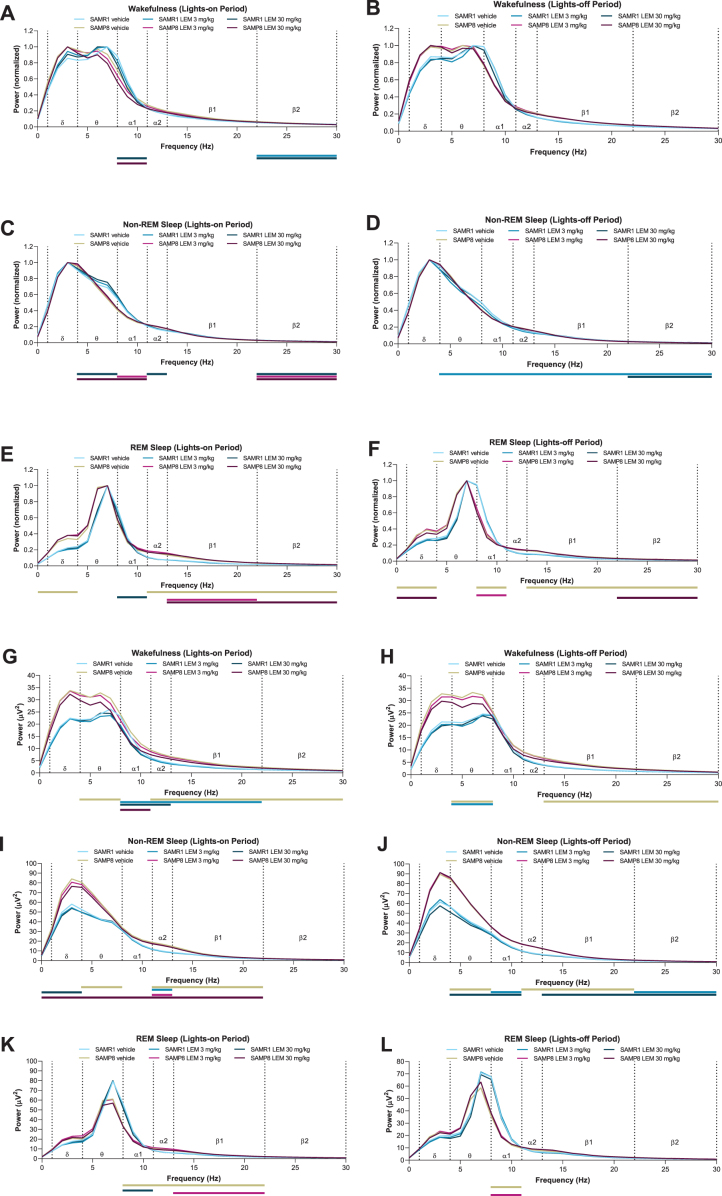
Effect of lemborexant (LEM) on A–F) normalized and G–L) absolute electroencephalogram (EEG) power spectra in senescence-accelerated mouse prone-8 (SAMP8) and senescence-accelerated mouse resistant-1 (SAMR1) mice. In a randomized crossover design, mice (SAMR1, *n* = 7 per treatment; SAMP8, *n* = 8 per treatment) received single oral doses of vehicle, LEM 3 mg/kg, and LEM 30 mg/kg at Zeitgeber time 0:00–0:30. EEG spectra bands (designated by dotted vertical lines) are as follows: *δ*= 1–4 Hz; *θ*= 4–8 Hz; α1 = 8–11 Hz; α2 = 11–13 Hz; β1 = 13–22 Hz; β2 = 22–30 Hz. Data are mean values. REM, rapid eye movement. Gold bar indicates *p* < 0.05 versus SAMR1 vehicle for EEG bands. Other bars indicate *p* < 0.05 versus vehicle within strain. Statistical analyses were performed using linear mixed-model analysis (with treatment/strain as fixed effects and animal and spectra band as a random effect), followed by Fisher’s least significance difference test (SAMR1-vehicle versus SAMP8-vehicle) or Dunnett type multiple comparison test (versus vehicle).

When analyzing normalized EEG power spectra, spectrum peaks during wakefulness in vehicle-treated SAMP8 mice appeared to have shifted to lower frequencies in both periods compared with vehicle-treated SAMR1 mice (Figs. 5A, B). Vehicle-treated SAMP8 mice also showed some differences in power bands during REM sleep compared with vehicle-treated SAMR1 mice (Figs. 5E, F; Supplementary Table 1); however, there were no differences during non-REM sleep (Fig. 5C, D; Supplementary Table 1). As the slowing of wake EEG would be of particular interest for the lights-out period, we performed a statistical analysis of normalized EEG power spectra for every 3-h bin of the lights-out period (Supplementary Table 2). SAMP8 mice did not show any difference in power bands compared with SAMR1 mice, except for the alpha-1 band. There was no significant difference in peak frequency, although SAMP8 mice appeared to have lower peak frequencies than SAMR1 mice for every 3-h bin. Lemborexant had nearly no influence on wake band power and no influence on peak frequencies (Fig. 5A, 5B; Supplementary Table 2).

When looking at absolute power spectra, SAMP8 mice had clearly higher EEG power during wakefulness and non-REM sleep than SAMR1 mice (Fig. 5G–J; Supplementary Table 3), whereas differences were smaller between strains during REM sleep (Fig. 5K, L; Supplementary Table 3). Of note, delta band power during wakefulness was numerically higher in SAMP8 mice compared with SAMR1 mice in the lights-on period (Supplementary Table 3; 27.5μV^2^ for SAMP8 and 17.7μV^2^ for SAMR1; *p* = 0.056; point estimate, 9.6; 95%confidence interval [CI], –0.3 to 19.6) and during the lights-off period (Supplementary Table 3; 27.4μV^2^ for SAMP8 and 16.8μV^2^ for SAMR1; *p* = 0.101; point estimate, 10.5; 95%CI, –2.3 to 23.2). Lemborexant treatment slightly, but significantly, reduced some power bands during wakefulness and non-REM sleep in both strains (Fig. 5G–J; Supplementary Table 3), but did not have an influence on delta band power during wakefulness in SAMP8 mice (Fig. 5G, H; Supplementary Table 3).

### Effect of lemborexant on diurnal rhythm of running wheel activity in SAMP8 and\\ SAMR1 mice

**Fig. 6:**
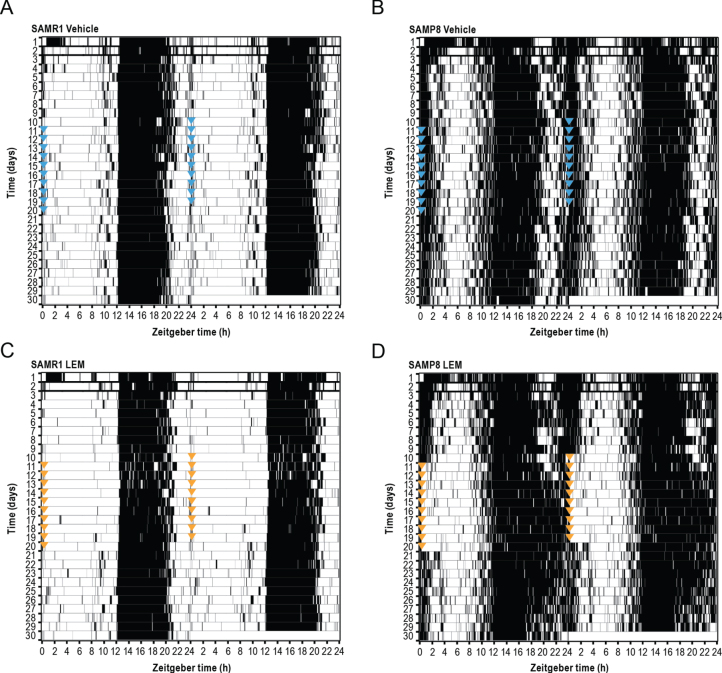
Effect of lemborexant (LEM) on normalized running wheel activity depicted as double-plotted actograms in A, C) senescence-accelerated mouse resistant-1 (SAMR1) and B, D) senescence-accelerated mouse prone-8 (SAMP8) mice. Mice (*n* = 6 per strain) were assessed for 10 days without dosing, of which days 3–10 were used for analysis (baseline) and then dosed orally with vehicle (blue arrow heads) or LEM 30 mg/kg (orange arrow heads) at the onset of light (Zeitgeber time [ZT] 0:00–0:30) each day for a further 10 days. Lights were on from ZT 0–12 each day. Posttreatment phase assessments (no LEM dosing) were then performed for 10 days. Each running bout is represented by a black line and no significant running wheel activity is represented by white coloring.

**Fig. 7:**
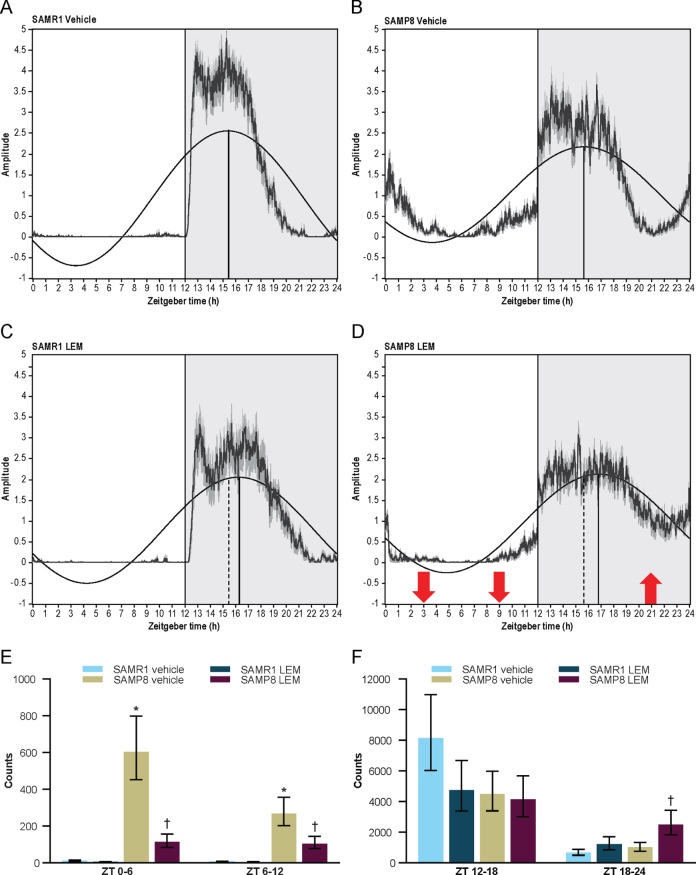
Averaged 24-h running wheel activity in vehicle and lemborexant (LEM) 30 mg/kg–treated A, C, E, F) senescence-accelerated mouse resistant-1 (SAMR1) mice (*n* = 12) and B, D, E, F) senescence-accelerated mouse prone-8 (SAMP8) mice (*n* = 11–12) during the treatment phase of the study. Pretreatment assessments were performed for 10 days without dosing, of which days 3–10 were used for analysis. Mice were then dosed orally with vehicle or LEM 30 mg/kg at the onset of light (Zeitgeber time [ZT] 0:00–0:30) each day for a further 10 days during the treatment phase. The shaded area indicates the lights-off period and the vertical line indicates acrophase (dotted vertical lines in C and D represent acrophase for corresponding vehicle-treated mice [A and B, respectively]). Red arrows indicate a significant within-strain difference (*p* < 0.05) between LEM and vehicle for 6-h bins (ZT 0–6, 6–12, and 18–24). ^*^p < 0.05 versus SAMR1 vehicle. ^†^*p* < 0.05 versus SAMP8 vehicle. Statistical analyses were performed using unpaired *t*-test.

SAMR1 mice had a clearly defined diurnal pattern of running wheel activity, with minimal activity during the lights-on period, activity increasing just after lights out (cohort average ZT 12.5), and activity decreasing approximately 3-h before the end of the lights-off period (ZT 21; Figs. 6A and 7A). In contrast, SAMP8 mice had an abnormal diurnal pattern of running wheel activity, which was characterized by the onset of activity before the start of the lights-off period (cohort average ZT 10.6), a decrease in activity in the latter half of the lights-off period (ZT 18–22), and a second peak of activity beginning late in the lights-off period, which extended well into the lights-on period (ZT 22–4; Figs. 6B and 7B). This finding is in keeping with a previous report [18]. Vehicle treatment had no apparent effect on activity levels or patterns in either mouse strain (Supplementary Figure 4A, B, G, H). Treatment with lemborexant 30 mg/kg at ZT 0 did not change running wheel pattern or activity counts in SAMR1 mice (Figs. 6C and 7C; Supplementary Figure 4E; Supplementary Table 4). However, lemborexant-treated SAMP8 mice displayed decreased activity during the first few hours and last few hours of the lights-on period, and increased activity in the latter half of the lights-off period (Figs. 6D and 7D; Supplementary Fig. 4K; Supplementary Table 4). Importantly, this lemborexant-induced consolidation of the diurnal light-dark activity rhythms was lost during a posttreatment washout phase (Supplementary Table 4; Supplementary Figure 4L). Quantification of total activity counts within 6-h time bins again revealed strikingly higher activity during the lights-on period (ZT 0–6 and ZT 6–12) in SAMP8 mice. This was apparent during the pretreatment phase in all mice (Supplementary Table 5; Supplementary Figure 5), as well as in vehicle-treated mice (Fig. 7E). Lemborexant treatment significantly reduced activity in SAMP8 mice during the lights-on period (ZT 0–6 and 6–12), with a consequent increase in activity during the latter half of the lights-off period (ZT 18–24; Fig. 7D–F; Supplementary Table 4). Lemborexant induced a significant delay in the acrophase of activity in both SAMR1 and SAMP8 mice, likely by indirectly promoting activity during the ZT 18–24 period, although the increase in activity counts did not reach statistical significance in SAMR1 mice (Fig. 7C, D; Supplementary Table 4). No changes in nonparametric measures of circadian fragmentation, IV and IS, were observed in SAMP8 mice (Supplementary Table 5) or following lemborexant treatment (Supplementary Table 4).

## DISCUSSION

Overall, SAMP8 mice display some, but not all, aspects of sleep-wake disturbances in AD. Whereas SAMP8 mice do not show sleep-wake fragmentation or increased sleep during the lights-off (active) period, they do display higher activity and less sleep during the lights-on (inactive) period, which is similar to the higher nighttime activity in patients with AD. Although no AD mouse model exhibits all aspects of sleep-wake disturbances in AD [20], SAMP8 mice show this important feature. Notably, lemborexant treatment ameliorated this aberrant activity during the lights-on period, while also increasing wakefulness during the lights-off period, suggesting possible utility in treating these critical aspects of sleep-wake disturbance in AD.

### PK of lemborexant in SAMP8 and SAMR1 mice

Following a single oral administration, lemborexant was rapidly absorbed in both strains, with plasma concentrations peaking at 0.25-h and decreasing to approximately 0.05%of C_max_ at 24-h post dose. The shape of the plasma concentration-time profiles and plasma concentrations at 3-h and thereafter were similar between strains. Thus, the difference in AUC from time 0–24-h (1.7-fold) between strains can be attributed to the difference in plasma concentration in the early period after administration (approximately 2-h). Plasma concentrations in both strains during this period were much higher than the concentration assumed necessary for a sleep-inducing effect, which can be estimated from K_i_ values for murine orexin receptors (8.3 nmol/L for orexin-1 receptor and 0.64 nmol/L for orexin-2 receptor [21]) and plasma protein binding (85.2%[32]). Assuming that the unbound plasma concentration is equal to the unbound concentration at the target site in the brain, the unbound concentration of lemborexant at the target site in SAMR1 and SAMP8 mice at 3-h after dosing is a minimum of 14.3 ng/mL, which is 34.8 nmol/L (molecular weight 410.4  g/mol). Due to the lag time needed for the distribution to the brain, the unbound concentration in the brain might not be equivalent to the unbound plasma concentration. However, considering that the CSF concentration of lemborexant reached steady state in mice 1-h after oral administration [32], rapid distribution to the brain is expected. Therefore, the observed difference in plasma concentration is not anticipated to result in a difference in pharmacological effect between strains.

### Diurnal orexin concentrations and effect of lemborexant on diurnal orexin concentrations in SAMP8 and SAMR1 mice

Our evaluation of diurnal orexin concentrations revealed that the diurnal variation of CSF OXA in vehicle-treated SAMR1 mice is consistent with previous reports in mice [33] and is as expected for a wake-promoting neuropeptide in nocturnal animals. In contrast, vehicle-treated SAMP8 mice had maximal OXA concentrations during the lights-on period, with clearly higher levels than control SAMR1 mice during the lights-on period, corresponding with higher wakefulness and running wheel activity. In contrast, CSF OXA levels were similar between the strains in the lights-off period, with no difference in wakefulness time. This finding of higher CSF OXA levels in SAMP8 during the lights-on period is noteworthy given that lumbar CSF OXA levels 1–1.5-h after waking in patients with mild-to-moderate AD correlate with the severity of their sleep disturbances [34]. As there is a time lag of several hours between labeling of amino acids and the capacity to detect this labeling in lumbar CSF in humans [35], presumably, morning lumbar CSF OXA levels in patients with AD reflect events of the night before. This relationship may be considered analogous to the aforementioned relationship between CSF OXA levels in SAMP8 mice and wakefulness/activity.

Patients with AD exhibit extensive loss of orexin neurons in the lateral hypothalamus/perifornical nuclei due to accumulation of tau [36]. Therefore, the higher CSF OXA levels in patients with AD appear surprising. However, other wake-promoting neurons, namely noradrenergic neurons in the locus coeruleus and histaminergic neurons in the tuberomammillary nucleus, are also greatly diminished in patients with AD. Given that noradrenergic neurons in the locus coeruleus (as well as serotonergic neurons in the Raphe nuclei) form a negative feedback loop with orexin neurons [37, 38], decreased inhibition of these orexin neurons may lead to higher activity in the remaining orexin neurons, resulting in higher OXA concentrations. Loss of neurons providing inhibitory feedback to orexin neurons, such as noradrenergic or serotonergic neurons, may also occur in SAMP8 mice, which would explain the higher OXA concentration over the murine rest period. Additional studies are needed to examine this possibility.

Our finding that lemborexant increased OXA levels by approximately two-fold in both strains cannot currently be explained, and has, to the best of our knowledge, not previously been described, and therefore warrants further investigation. Nevertheless, despite this increase in OXA concentration, both strains showed increased sleep behavior with lemborexant treatment during the lights-on period, suggesting that at sufficiently high concentrations, lemborexant is competing effectively for the orexin receptors and is able to promote sleep, even in the presence of elevated OXA levels. The fact that sleep is promoted in the presence of increased OXA concentrations might be due to lemborexant blocking the majority of orexin receptors, which is in alignment with the PK data, leaving secreted OXA unable to bind to the receptors and causing accumulation of OXA in the CSF.

### Effect of lemborexant on vigilance states in SAMP8 and SAMR1 mice

An important finding of our evaluation of vigilance states is that, under vehicle treatment conditions, SAMP8 mice showed higher levels of wakefulness during the lights-on period (the rest period for mice) compared with SAMR1 mice. It therefore appears that SAMP8 mice display the equivalent of sleep disturbances during the rest period, which is likely explained by higher CSF OXA levels. Lemborexant dose-dependently decreased the level of wakefulness in both strains during the lights-on period, with corresponding increases in non-REM and REM sleep times observed. Of note, lemborexant increased levels of wakefulness in SAMP8, but not SAMR1, mice during the lights-off period (the active period for mice). Although total wakefulness time over the diurnal cycle was not changed, diurnal wake ratio calculations indicated that lemborexant shifts wakefulness from the lights-on period to the lights-off period. One of the aims of treating sleep-wake disturbances in humans with AD is promoting sleep at night and increasing or consolidating next-day wakefulness; in other words, reducing the frequency and/or duration of sleep bouts. SAMP8 mice did not display a wakefulness deficit during the lights-off (active) period compared with control SAMR1 mice, as measured by wake/sleep time or wake EEG peak frequency, and therefore, did not exhibit this important daytime aspect of sleep-wake disturbance of human AD. However, SAMP8 mice reacted to lemborexant treatment with increased wakefulness, which is analogous to the desired clinical outcome in humans. As already mentioned, the precise reasons underlying this finding are yet to be elucidated; however, a direct effect of orexin receptor antagonism seems unlikely based on the plasma concentration profile of the drug. Off-target activity also appears unlikely given that the orexin receptor selectivity of lemborexant is≥1,700-fold compared with other targets [21].

The shorter REM sleep time in SAMP8 mice compared with SAMR1 mice during the lights-on period may also be due to higher CSF OXA concentrations in these mice. Orexin neurons have been shown to inhibit melanin-concentrating hormone neurons in the lateral hypothalamic area, which are responsible for REM sleep onset [38] and fire nearly exclusively during REM sleep [39, 40]. Therefore, increased OXA levels would suppress melanin-concentrating hormone neuronal activity and hamper REM sleep promotion. Whether this is the case in SAMP8 mice and what effects lemborexant has on the OXA/melanin-concentrating hormone feedback loop remains to be determined.

Consistent with vigilance state findings, SAMP8 mice showed numerically longer sleep latency and REM sleep latency compared with SAMR1 mice, while lemborexant reduced sleep latency and REM sleep latency in both strains of mice, a finding that has also been observed in humans [24–26, 41]. Of note, we found that sleep latency was similar between strains following treatment with lemborexant.

Our analysis of normalized EEG power spectra revealed that the wakefulness EEG spectra peaks of SAMP8 mice were shifted to numerically lower frequencies in both periods compared with SAMR1 mice, although this finding did not reach statistical significance. This finding is consistent with those obtained using the PLB1Triple mouse (hAPP/hTau/hPS1) AD model [42]. The slowing of wake EEG is a hallmark of AD and likely reflects failing synchronization and connectivity between brain regions [42–44]. Lemborexant had no influence on normalized EEG frequency distribution in either strain, regardless of vigilance state. However, when analyzing non-normalized EEG power spectra data, SAMP8 mice had clearly higher EEG power than SAMR1 mice during wakefulness and non-REM sleep, but not during REM sleep. This indicates a vigilance state-dependent difference between the strains. The numerically higher delta power in SAMP8 mice during wakefulness (Supplementary Table 3) resembles what has been described for patients with AD, who have increased delta power compared with healthy controls [45].

We found that SAMP8 mice had comparatively less REM sleep in the lights-on period, resulting in an overall reduction in REM sleep time. In patients with AD, REM sleep is decreased due to reduced REM bout duration, while occurrence is unchanged [46]. In our studies, there was no significant indication of increased fragmentation of sleep-wake behavior in SAMP8 mice. However, during the lights-on period, lemborexant caused some increased fragmentation in both strains, which is less desirable. On the other hand, the observed increase in wake time with lemborexant in SAMP8 mice during the lights-off period seems to be caused by a reduction in the wake bout count and a dose-dependent increase in wake bout duration; in other words, by consolidation of the wakefulness state during the active period, which would be a desired clinical feature.

Lemborexant 30 mg/kg was associated with sporadic SOREM events in three SAMP8 mice; such events were also observed in one vehicle-treated SAMP8 mouse. In a previous report, lemborexant was found to cause SOREM events at comparable doses in mice when combined with a strong emotional feeding stimulus [23]. However, we believe that these findings do not suggest serious safety concerns, particularly in light of previous preclinical and clinical experience with lemborexant [22, 24–26, 47, 48].

### Effect of lemborexant on diurnal rhythm of running wheel activity in SAMP8 and\\ SAMR1 mice

Overall, the findings from the running wheel actigraphy studies aligned well with findings from the sleep-wake measurements. Running wheel actigraphy data demonstrated an altered diurnal pattern of activity in SAMP8 mice compared with SAMR1 mice, with significantly more activity during the lights-on period (Figs. 6B and 7B) and numerically less activity during the lights-off period. This pattern is reminiscent of patients with AD, who may show less activity during the day and heightened activity in the evening and at night. Lemborexant helped to normalize this pattern by decreasing activity during the lights-on period and increasing activity in the latter half of the lights-off period, thereby consolidating activity in the appropriate light-dark period. If these findings were to translate to humans, we would expect that lemborexant, given at bedtime, could not only consolidate nighttime rest/sleep, but also increase daytime activity in patients with AD who experience sleep-wake disturbances. This normalization of diurnal activity patterns could be highly beneficial for patients with AD who experience sleep-wake disturbances; human studies are pending. Lemborexant did delay the acrophase of running wheel activity by approximately 1-h in both SAMR1 and SAMP8 mice, an effect which would be undesirable in humans with AD who already exhibit phase delay. However, this effect is likely due to the increase in activity in the latter part of the active phase, as both mouse strains show a lull in activity in the middle of the dark phase, which is not observed in humans. Moreover, consolidation of activity during the active phase and reduction of unwanted activity during the rest phase is a desirable potential clinical effect. Notably, lemborexant did not reduce total activity counts in either mouse strain, suggesting there was no persisting sedative effect during the active phase.

Interestingly, lemborexant increased activity in SAMP8 mice during the ZT 18–24 period, despite the fact that lemborexant plasma concentrations had decreased to≤2.57 ng/mL. This is equivalent to a≤0.93 nmol/L unbound concentration in the brain, and therefore around or below K_i_ values of lemborexant for orexin receptors by that time post injection (see Fig. 1). For this reason, and because it is very difficult to imagine that antagonism of the wake-promoting orexin receptors would directly promote wakefulness, this effect in the latter portion of the lights-off period may be indirect, perhaps due to a carryover effect from increased sleep during the lights-on period, or an effect on circadian rhythms. As these experiments were carried out under light-dark conditions, direct conclusions about circadian effects of lemborexant cannot be made. Further studies examining the effects of lemborexant on the circadian system are needed.

## CONCLUSIONS

Compared with SAMR1 mice, SAMP8 mice showed sleep-wake and diurnal rhythm aberrances, most notably more wakefulness/activity during the resting (lights-on) period, a tendency to slowed wake EEG, and a tendency to higher delta power during wakefulness. Other aspects of sleep-wake disturbances in AD, such as rhythm fragmentation and phase delay, were not observed. Previous studies have also shown that SAMP8 mice have memory/cognition deficits and brain pathologies resembling human AD [17]. Taken together, these findings suggest that SAMP8 mice display some sleep-wake and diurnal rhythm abnormalities observed in patients with AD, in particular increased nighttime activity and wakefulness.

We found that many aberrances in SAMP8 mice could be modified by treatment with the DORA lemborexant. Not only did lemborexant reduce activity and increase sleep during the resting period, as expected from an insomnia drug, but also resulted in higher levels of wakefulness and activity during the active period, as would be necessary for treating sleep-wake disturbances in patients with AD. These findings provide some preclinical rationale for the clinical evaluation of lemborexant in patients with AD who suffer from sleep-wake disturbances. This report also provides preliminary evidence that a DORA can influence diurnal activity patterns.

## Supplementary Material

Supplementary Material
