# Supplementary material for: Evaluation of SAMP8 Mice as a Model for Sleep-Wake and Rhythm Disturbances Associated with Alzheimer’s Disease: Impact of Treatment with the Dual Orexin (Hypocretin) Receptor Antagonist Lemborexant
Source: J Alzheimers Dis. 2021 Jun 1;81(3):1151–67. doi: 10.3233/JAD-201054 (PMC8293654; doi:10.3233/JAD-201054)

Supplementary Material

Evaluation of SAMP8 Mice as a Model for Sleep-Wake and Rhythm Disturbances Associated with Alzheimer's Disease: Impact of Treatment with the Dual Orexin (Hypocretin) Receptor Antagonist Lemborexant

Supplementary Table 1. Effect of lemborexant on normalized EEG power spectra bands[a] in SAMP8 and SAMR1 mice[b]

	SAMR1			SAMP8		
**Vigilance state**<br>**EEG power spectra band[a]**	**Vehicle**<br>**(*n* = 7)**	**LEM 3 mg/kg**<br>**(*n* = 7)**	**LEM 30 mg/kg**<br>**(*n* = 7)**	**Vehicle**<br>**(*n* = 8)**	**LEM 3 mg/kg**<br>**(*n* = 8)**	**LEM 30 mg/kg**<br>**(*n* = 8)**
Wakefulness (lights-on)						
δ	0.677 ± 0.059	0.723 ± 0.059	0.703 ± 0.059	0.813 ± 0.104	0.807 ± 0.104	0.791 ± 0.104
θ	0.899 ± 0.069	0.945 ± 0.069	0.939 ± 0.069	0.938 ± 0.066	0.916 ± 0.066	0.869 ± 0.066
α1	0.600 ± 0.057	0.564 ± 0.057	0.519 ± 0.057*	0.520 ± 0.039	0.467 ± 0.039	0.407 ± 0.039*
α2	0.209 ± 0.015	0.211 ± 0.015	0.206 ± 0.015	0.252 ± 0.024	0.236 ± 0.024	0.215 ± 0.024
β1	0.095 ± 0.007	0.098 ± 0.007	0.097 ± 0.007	0.124 ± 0.012	0.119 ± 0.012	0.110 ± 0.012
β2	0.041 ± 0.002	0.044 ± 0.002*	0.044 ± 0.002*	0.048 ± 0.005	0.047 ± 0.005	0.045 ± 0.005
Wakefulness (lights-off)						
δ	0.684 ± 0.063	0.657 ± 0.063	0.660 ± 0.063	0.823 ± 0.129	0.814 ± 0.129	0.828 ± 0.129
θ	0.910 ± 0.067	0.880 ± 0.067	0.906 ± 0.067	0.969 ± 0.081	0.983 ± 0.081	0.957 ± 0.081
α1	0.709 ± 0.089	0.714 ± 0.089	0.660 ± 0.089	0.549 ± 0.043	0.565 ± 0.043	0.553 ± 0.043
α2	0.235 ± 0.018	0.229 ± 0.018	0.224 ± 0.018	0.254 ± 0.029	0.262 ± 0.029	0.244 ± 0.029
β1	0.100 ± 0.007	0.097 ± 0.007	0.099 ± 0.007	0.129 ± 0.015	0.131 ± 0.015	0.129 ± 0.015
β2	0.043 ± 0.002	0.043 ± 0.002	0.044 ± 0.002	0.051 ± 0.005	0.053 ± 0.005	0.053 ± 0.005
Non-REM sleep (lights-on)						
δ	0.774 ± 0.073	0.781 ± 0.073	0.771 ± 0.073	0.742 ± 0.085	0.742 ± 0.085	0.737 ± 0.085
θ	0.781 ± 0.068	0.806 ± 0.068	0.831 ± 0.068*	0.755 ± 0.065	0.771 ± 0.065	0.784 ± 0.065*
α1	0.400 ± 0.035	0.413 ± 0.035	0.421 ± 0.035	0.322 ± 0.028	0.330 ± 0.028*	0.336 ± 0.028*
α2	0.187 ± 0.013	0.191 ± 0.013	0.195 ± 0.013*	0.212 ± 0.019	0.210 ± 0.019	0.215 ± 0.019
β1	0.081 ± 0.006	0.083 ± 0.006	0.085 ± 0.006	0.083 ± 0.008	0.084 ± 0.008	0.085 ± 0.008
β2	0.022 ± 0.001	0.022 ± 0.001	0.023 ± 0.001*	0.019 ± 0.002	0.020 ± 0.002*	0.020 ± 0.002*

Non-REM sleep (lights-off)						
δ	0.772 ± 0.070	0.768 ± 0.070	0.768 ± 0.070	0.731 ± 0.087	0.731 ± 0.087	0.723 ± 0.087
θ	0.741 ± 0.054	0.694 ± 0.054*	0.725 ± 0.054	0.734 ± 0.061	0.722 ± 0.061	0.729 ± 0.061
α1	0.370 ± 0.030	0.338 ± 0.030*	0.369 ± 0.030	0.317 ± 0.027	0.314 ± 0.027	0.313 ± 0.027
α2	0.176 ± 0.012	0.165 ± 0.012*	0.180 ± 0.012	0.196 ± 0.018	0.191 ± 0.018	0.193 ± 0.018
β1	0.077 ± 0.006	0.072 ± 0.006*	0.078 ± 0.006	0.079 ± 0.008	0.078 ± 0.008	0.077 ± 0.008
β2	0.021 ± 0.001	0.019 ± 0.001*	0.022 ± 0.001*	0.018 ± 0.002	0.018 ± 0.002	0.018 ± 0.002
REM sleep (lights-on)						
δ	0.168 ± 0.014	0.159 ± 0.014	0.163 ± 0.014	0.266 ± 0.022†	0.287 ± 0.022	0.287 ± 0.022
θ	0.551 ± 0.061	0.543 ± 0.061	0.561 ± 0.061	0.695 ± 0.053	0.716 ± 0.053	0.709 ± 0.053
α1	0.412 ± 0.053	0.403 ± 0.053	0.372 ± 0.053*	0.345 ± 0.023	0.363 ± 0.023	0.367 ± 0.023
α2	0.096 ± 0.009	0.095 ± 0.009	0.093 ± 0.009	0.163 ± 0.013†	0.180 ± 0.013	0.164 ± 0.013
β1	0.046 ± 0.003	0.043 ± 0.003	0.044 ± 0.003	0.079 ± 0.006†	0.086 ± 0.006*	0.086 ± 0.006*
β2	0.014 ± 0.001	0.014 ± 0.001	0.013 ± 0.001	0.024 ± 0.002†	0.024 ± 0.002	0.026 ± 0.002*
REM sleep (lights-off)						
δ	0.210 ± 0.018	0.194 ± 0.018	0.204 ± 0.018	0.288 ± 0.021†	0.286 ± 0.021	0.260 ± 0.021*
θ	0.536 ± 0.045	0.524 ± 0.045	0.511 ± 0.045	0.661 ± 0.051	0.666 ± 0.051	0.641 ± 0.051
α1	0.556 ± 0.080	0.564 ± 0.080	0.556 ± 0.080	0.377 ± 0.026†	0.402 ± 0.026*	0.367 ± 0.026
α2	0.122 ± 0.013	0.119 ± 0.013	0.123 ± 0.013	0.153 ± 0.013	0.161 ± 0.013	0.155 ± 0.013
β1	0.058 ± 0.004	0.054 ± 0.004	0.056 ± 0.004	0.081 ± 0.007†	0.083 ± 0.007	0.081 ± 0.007
β2	0.015 ± 0.001	0.015 ± 0.001	0.016 ± 0.001	0.025 ± 0.002†	0.025 ± 0.002	0.022 ± 0.002*

Data are mean ± standard error of the mean.

[a] EEG spectra bands are as follows: δ = 1–4 Hz; θ = 4–8 Hz; α1 = 8–11 Hz; α2 = 11–13 Hz; β1 = 13–22 Hz; β2 = 22–30 Hz.

[b] Mice received single oral doses at Zeitgeber time 0:00–0:30.

*$p < 0.05$ versus vehicle; †$p < 0.05$ versus vehicle-treated SAMR1.

EEG, electroencephalogram; LEM, lemborexant; REM, rapid eye movement; SAMP8, senescence-accelerated mouse prone-8; SAMR1, senescence-accelerated mouse resistant-1.

Statistical analyses for EEG band power spectra analysis data were performed using linear mixed-model analysis (with treatment/strain as fixed effects and animal and spectra band as a random effect) followed by Fisher's least significance difference test (SAMR1 versus SAMP8) or Dunnett type multiple comparison test (versus vehicle).

Supplementary Table 2. Effect of lemborexant on normalized wakefulness EEG power spectra bands[a] and peak frequencies in SAMP8 and SAMR1 mice[b] during lights-off (in 3-h bins)

	**SAMR1**			**SAMP8**		
**Vigilance state**<br>**EEG power spectra band[a]**	**Vehicle (*n* = 7)**	**LEM 3 mg/kg (*n* = 7)**	**LEM 30 mg/kg (*n* = 7)**	**Vehicle (*n* = 8)**	**LEM 3 mg/kg (*n* = 8)**	**LEM 30 mg/kg (*n* = 8)**
Wakefulness (ZT12–15)						
δ	0.663 ± 0.041	0.667 ± 0.041	0.636 ± 0.041	0.671 ± 0.049	0.690 ± 0.049	0.733 ± 0.049
θ	0.872 ± 0.033	0.850 ± 0.033	0.875 ± 0.033	0.867 ± 0.022	0.876 ± 0.022	0.906 ± 0.022
α1	0.640 ± 0.034	0.557 ± 0.034*	0.549 ± 0.034*	0.520 ± 0.025†	0.508 ± 0.025	0.481 ± 0.025
α2	0.240 ± 0.018	0.219 ± 0.018	0.213 ± 0.018*	0.235 ± 0.015	0.241 ± 0.015	0.237 ± 0.015
β1	0.100 ± 0.008	0.093 ± 0.008	0.096 ± 0.008	0.122 ± 0.007	0.121 ± 0.007	0.125 ± 0.007
β2	0.043 ± 0.004	0.041 ± 0.004	0.043 ± 0.004	0.049 ± 0.004	0.051 ± 0.004	0.051 ± 0.004
peak frequency (Hz)	5.84 ± 0.65	5.28 ± 0.65	6.16 ± 0.65	5.12 ± 0.67	4.97 ± 0.67	5.15 ± 0.67
Wakefulness (ZT15–18)						
δ	0.634 ± 0.047	0.633 ± 0.047	0.649 ± 0.047	0.715 ± 0.062	0.712 ± 0.062	0.724 ± 0.062
θ	0.836 ± 0.035	0.823 ± 0.035	0.854 ± 0.035	0.840 ± 0.020	0.881 ± 0.020	0.849 ± 0.020
α1	0.653 ± 0.044	0.651 ± 0.044	0.597 ± 0.044	0.512 ± 0.024†	0.542 ± 0.024	0.569 ± 0.024*
α2	0.220 ± 0.020	0.218 ± 0.020	0.218 ± 0.020	0.218 ± 0.017	0.233 ± 0.017	0.223 ± 0.017
β1	0.095 ± 0.009	0.095 ± 0.009	0.099 ± 0.009	0.111 ± 0.009	0.117 ± 0.009	0.120 ± 0.009
β2	0.041 ± 0.004	0.042 ± 0.004	0.044 ± 0.004	0.047 ± 0.004	0.046 ± 0.004	0.050 ± 0.004
peak frequency (Hz)	5.99 ± 0.78	5.28 ± 0.78	5.59 ± 0.78	4.87 ± 0.72	5.60 ± 0.72	5.03 ± 0.72
Wakefulness (ZT18–21)						
δ	0.631 ± 0.060	0.572 ± 0.060	0.629 ± 0.060	0.688 ± 0.053	0.701 ± 0.053	0.747 ± 0.053
θ	0.829 ± 0.050	0.788 ± 0.050	0.860 ± 0.050	0.894 ± 0.024	0.836 ± 0.024	0.829 ± 0.024
α1	0.656 ± 0.020	0.683 ± 0.020	0.667 ± 0.020	0.461 ± 0.037†	0.457 ± 0.037	0.477 ± 0.037
α2	0.214 ± 0.019	0.220 ± 0.019	0.215 ± 0.019	0.220 ± 0.019	0.219 ± 0.019	0.204 ± 0.019
β1	0.093 ± 0.011	0.093 ± 0.011	0.097 ± 0.011	0.112 ± 0.010	0.105 ± 0.010	0.108 ± 0.010
β2	0.042 ± 0.005	0.041 ± 0.005	0.043 ± 0.005	0.044 ± 0.004	0.041 ± 0.004	0.043 ± 0.004
peak frequency (Hz)	6.84 ± 0.60	6.85 ± 0.60	7.02 ± 0.60	5.12 ± 0.68	4.47 ± 0.68	3.90 ± 0.68
Wakefulness (ZT21–24)						
δ	0.618 ± 0.058	0.590 ± 0.058	0.612 ± 0.058	0.712 ± 0.049	0.729 ± 0.049	0.724 ± 0.049

θ	0.861 ± 0.042	0.825 ± 0.042	0.859 ± 0.042	0.881 ± 0.027	0.910 ± 0.027	0.854 ± 0.027
α1	0.580 ± 0.022	0.618 ± 0.022	0.623 ± 0.022	0.477 ± 0.027†	0.478 ± 0.027	0.451 ± 0.027
α2	0.200 ± 0.019	0.192 ± 0.019	0.214 ± 0.019	0.230 ± 0.019	0.229 ± 0.019	0.203 ± 0.019
β1	0.094 ± 0.010	0.089 ± 0.010	0.094 ± 0.010	0.115 ± 0.010	0.116 ± 0.010	0.108 ± 0.010
β2	0.041 ± 0.004	0.039 ± 0.004	0.043 ± 0.004	0.045 ± 0.004	0.045 ± 0.004	0.045 ± 0.004
peak frequency (Hz)	6.27 ± 0.63	6.57 ± 0.63	6.59 ± 0.63	4.37 ± 0.58	4.97 ± 0.58	4.28 ± 0.58

Data are mean ± standard error of the mean.

[a] EEG spectra bands are as follows: δ = 1–4 Hz; θ = 4–8 Hz; α1 = 8–11 Hz; α2 = 11–13 Hz; β1 = 13–22 Hz; β2 = 22–30 Hz.

[b] Mice received single oral doses at Zeitgeber time 0:00–0:30.

*$p < 0.05$ versus vehicle; †$p < 0.05$ versus vehicle-treated SAMR1.

EEG, electroencephalogram; LEM, lemborexant; SAMP8, senescence-accelerated mouse prone-8; SAMR1, senescence-accelerated mouse resistant-1.

Statistical analyses for EEG band power spectra analysis data were performed using linear mixed-model analysis (with treatment/strain as fixed effects and animal and spectra band as a random effect), followed by Fisher's least significance difference test (SAMR1-vehicle versus SAMP8-vehicle) or Dunnett type multiple comparison test (versus vehicle). Statistical analyses for peak frequency were performed using linear mixed-model analysis (with treatment/strain as fixed effects and animal and time-bin as a random effect), followed by Fisher's least significance difference test (SAMR1-vehicle versus SAMP8-vehicle) or Dunnett type multiple comparison test (versus vehicle).

Supplementary Table 3. Effect of lemborexant on absolute EEG power spectra bands[a] in SAMP8 and SAMR1 mice[b]

**Vigilance state**	**SAMR1**			**SAMP8**		
**EEG power spectra band[a]**	**Vehicle (*n* = 7)**	**LEM 3 mg/kg (*n* = 7)**	**LEM 30 mg/kg (*n* = 7)**	**Vehicle (*n* = 8)**	**LEM 3 mg/kg (*n* = 8)**	**LEM 30 mg/kg (*n* = 8)**
Wakefulness (lights-on)						
δ	17.7 ± 1.5	17.0 ± 1.5	17.2 ± 1.5	27.5 ± 3.5	27.1 ± 3.5	25.5 ± 3.5
θ	23.5 ± 1.7	22.2 ± 1.7	23.0 ± 1.7	31.7 ± 2.2†	30.8 ± 2.2	28.1 ± 2.2
α1	15.7 ± 1.4	13.3 ± 1.4*	12.7 ± 1.4*	17.6 ± 1.3	15.7 ± 1.3	13.1 ± 1.3*
α2	5.5 ± 0.4	5.0 ± 0.4*	5.0 ± 0.4*	8.5 ± 0.8†	7.9 ± 0.8	6.9 ± 0.8
β1	2.5 ± 0.2	2.3 ± 0.2*	2.4 ± 0.2	4.2 ± 0.4†	4.0 ± 0.4	3.6 ± 0.4
β2	1.1 ± 0.1	1.0 ± 0.1	1.1 ± 0.1	1.6 ± 0.2†	1.6 ± 0.2	1.5 ± 0.2
Wakefulness (lights-off)						
δ	16.8 ± 1.5	15.9 ± 1.5	15.7 ± 1.5	27.4 ± 4.1	25.8 ± 4.1	24.6 ± 4.1
θ	22.4 ± 1.6	21.3 ± 1.6*	21.6 ± 1.6	32.2 ± 2.6†	31.2 ± 2.6	28.5 ± 2.6
α1	17.4 ± 2.2	17.3 ± 2.2	15.7 ± 2.2	18.3 ± 1.4	17.9 ± 1.4	16.4 ± 1.4
α2	5.8 ± 0.4	5.5 ± 0.4	5.3 ± 0.4	8.4 ± 0.9	8.3 ± 0.9	7.2 ± 0.9
β1	2.5 ± 0.2	2.4 ± 0.2	2.4 ± 0.2	4.3 ± 0.5†	4.2 ± 0.5	3.8 ± 0.5
β2	1.1 ± 0.1	1.0 ± 0.1	1.0 ± 0.1	1.7 ± 0.2†	1.7 ± 0.2	1.6 ± 0.2
Non-REM sleep (lights-on)						
δ	45.0 ± 4.1	42.6 ± 4.1	41.5 ± 4.1*	62.4 ± 6.8	59.9 ± 6.8	56.3 ± 6.8*
θ	45.4 ± 3.7	43.9 ± 3.7	44.7 ± 3.7	63.5 ± 5.3†	62.3 ± 5.3	59.9 ± 5.3*
α1	23.3 ± 1.9	22.5 ± 1.9	22.6 ± 1.9	27.1 ± 2.3	26.7 ± 2.3	25.7 ± 2.3*
α2	10.9 ± 0.7	10.4 ± 0.7*	10.5 ± 0.7	17.8 ± 1.6†	16.9 ± 1.6*	16.4 ± 1.6*
β1	4.7 ± 0.4	4.6 ± 0.4	4.6 ± 0.4	7.0 ± 0.7†	6.7 ± 0.7	6.5 ± 0.7*
β2	1.3 ± 0.1	1.2 ± 0.1	1.2 ± 0.1	1.6 ± 0.1	1.6 ± 0.1	1.6 ± 0.1
Non-REM sleep (lights-off)						
δ	47.4 ± 4.3	49.1 ± 4.3	44.2 ± 4.3	65.4 ± 7.9	66.7 ± 7.9	66.0 ± 7.9
θ	45.5 ± 3.3	44.3 ± 3.3	41.7 ± 3.3*	65.7 ± 5.5†	65.9 ± 5.5	66.5 ± 5.5
α1	22.7 ± 1.8	21.6 ± 1.8*	21.2 ± 1.8*	28.4 ± 2.4	28.6 ± 2.4	28.6 ± 2.4
α2	10.8 ± 0.7	10.5 ± 0.7	10.4 ± 0.7	17.6 ± 1.6†	17.5 ± 1.6	17.6 ± 1.6
β1	4.8 ± 0.4	4.6 ± 0.4	4.5 ± 0.4*	7.0 ± 0.7†	7.1 ± 0.7	7.0 ± 0.7

β2	1.3 ± 0.1	1.2 ± 0.1*	1.2 ± 0.1*	1.6 ± 0.1	1.6 ± 0.1	1.6 ± 0.1
REM sleep (lights-on)						
δ	13.3 ± 1.1	12.7 ± 1.1	13.1 ± 1.1	16.2 ± 1.3	17.4 ± 1.3	16.4 ± 1.3
θ	43.6 ± 4.9	43.3 ± 4.9	45.0 ± 4.9	42.4 ± 3.1	43.4 ± 3.1	40.4 ± 3.1
α1	32.6 ± 4.2	32.1 ± 4.2	29.9 ± 4.2*	21.1 ± 1.3†	22.0 ± 1.3	20.9 ± 1.3
α2	7.6 ± 0.7	7.6 ± 0.7	7.4 ± 0.7	10.0 ± 0.8†	10.9 ± 0.8	9.4 ± 0.8
β1	3.6 ± 0.2	3.4 ± 0.2	3.5 ± 0.2	4.8 ± 0.4†	5.2 ± 0.4*	4.9 ± 0.4
β2	1.1 ± 0.1	1.1 ± 0.1	1.1 ± 0.1	1.5 ± 0.1	1.5 ± 0.1	1.5 ± 0.1
REM sleep (lights-off)						
δ	14.9 ± 1.3	13.9 ± 1.3	14.1 ± 1.3	16.9 ± 1.3	16.8 ± 1.3	16.5 ± 1.3
θ	38.1 ± 3.2	37.5 ± 3.2	35.5 ± 3.2	38.8 ± 3.0	39.1 ± 3.0	40.7 ± 3.0
α1	39.5 ± 5.7	40.4 ± 5.7	38.5 ± 5.7	22.1 ± 1.5†	23.6 ± 1.5*	23.3 ± 1.5
α2	8.7 ± 1.0	8.5 ± 1.0	8.5 ± 1.0	9.0 ± 0.8	9.4 ± 0.8	9.8 ± 0.8
β1	4.1 ± 0.3	3.8 ± 0.3	3.9 ± 0.3	4.8 ± 0.4	4.9 ± 0.4	5.1 ± 0.4
β2	1.1 ± 0.1	1.1 ± 0.1	1.1 ± 0.1	1.5 ± 0.1	1.5 ± 0.1	1.4 ± 0.1

Data are mean ± standard error of the mean μV^2.

[a] EEG spectra bands are as follows: δ = 1–4 Hz; θ = 4–8 Hz; α1 = 8–11 Hz; α2 = 11–13 Hz; β1 = 13–22 Hz; β2 = 22–30 Hz.

[b] Mice received single oral doses at Zeitgeber time 0:00–0:30.

*$p < 0.05$ versus vehicle within strain; †$p < 0.05$ versus vehicle-treated SAMR1.

EEG, electroencephalogram; LEM, lemborexant; REM, rapid eye movement; SAMP8, senescence-accelerated mouse prone-8; SAMR1, senescence-accelerated mouse resistant-1.

Statistical analyses for EEG band power spectra analysis data were performed using linear mixed-model analysis (with treatment/strain as fixed effects and animal and spectra band as random effects), followed by Fisher's least significance difference test (SAMR1-vehicle versus SAMP8-vehicle) or Dunnett type multiple comparison test (versus vehicle).

Supplementary Table 4. Diurnal running wheel activity in vehicle- and LEM 30 mg/kg–treated SAMP8 and SAMR1 mice during the pretreatment, treatment, and posttreatment phases of the study[a]

		SAMR1		SAMP8	
**Absolute activity/ 6 h**	**Phase**	**Vehicle (n = 12)**	**LEM 30 mg/kg (n = 12)**	**Vehicle (n = 11)**	**LEM 30 mg/kg (n = 12)**
ZT 0–6	Pretreatment	24 (18–32)	7 (5–10)*	532 (401–707)†	458 (333–629)
	Treatment	10 (8–14)	5 (3–7)	602 (453–799)†	114 (83–157)*
	Posttreatment	12 (9–16)	11 (8–16)	402 (303–534)†	414 (302–569)
ZT 6–12	Pretreatment	26 (19–35)	10 (7–14)*	385 (290–511)†	273 (198–374)
	Treatment	6 (5–9)	4 (3–6)	267 (201–355)†	104 (76–143)*
	Posttreatment	12 (9–16)	23 (16–32)	332 (250–441)†	302 (219–414)
ZT 12–18	Pretreatment	7037 (5216–9495)	5378 (3821–7568)	4779 (3597–6347)	4763 (3467–6543)
	Treatment	8128 (6024–10967)	4735 (3364–6663)	4498 (3386–5974)	4123 (3001–5664)
	Posttreatment	10937 (8106–14757)	9710 (6899–13665)	3801 (2862–5049)†	3939 (2867–5411)
ZT 18–24	Pretreatment	795 (589–1072)	1218 (866–1715)	1170 (881–1555)	1743 (1269–2394)
	Treatment	651 (483–879)	1202 (854–1692)	1000 (753–1328)	2496 (1817–3429)*
	Posttreatment	1755 (1300–2368)	1791 (1272–2520)	986 (742–1309)	1935 (1408–2658)
Intradaily variability	Treatment	0.49 ± 0.09	0.68 ± 0.09	0.78 ± 0.09	0.75 ± 0.09
Interdaily stability	Treatment	0.79 ± 0.04	0.61 ± 0.04*	0.67 ± 0.05	0.62 ± 0.04
Acrophase (ZT)	Treatment	15.4 ± 0.2	16.3 ± 0.2*	15.5 ± 0.3	16.8 ± 0.2*

Data are mean (standard error range) or ± standard error of the mean.

[a] Pretreatment assessments were performed for 10 days without dosing, of which days 3–10 were used for analysis. Mice were then dosed orally with vehicle or LEM 30 mg/kg at the onset of light (ZT 0:00–0:30) each day for a further 10 days during the treatment phase. Posttreatment phase assessments (no dosing) were then performed for 10 days.

*$p < 0.05$ versus vehicle within-strain comparison; †$p < 0.05$ versus vehicle-treated SAMR1 mice.

LEM, lemborexant; SAMP8, senescence-accelerated mouse prone-8; SAMR1, senescence-accelerated mouse resistant-1; ZT, Zeitgeber time.

Statistical analyses were performed using linear mixed-model analysis (with treatment/strain and time-bin as fixed effects and animal as a random effect), followed by Fisher's least significance difference test for log-transformed running wheel activity data.

Supplementary Table 5. Circadian running wheel activity in SAMP8 and SAMR1 mice during the pretreatment phase[a]

**Absolute activity/6 h**	**SAMR1 (*n* = 24)**	**SAMP8 (*n* = 23)**
ZT 0–6	13 (11–16)	494 (393–622)*
ZT 6–12	16 (13–20)	323 (257–407)*
ZT 12–18	6183 (4935–7747)	4792 (3805–6034)
ZT 18–24	989 (789–1239)	1447 (1149–1822)
Intradaily variability	0.63 ± 0.07	0.72 ± 0.06
Interdaily stability	0.71 ± 0.03	0.64 ± 0.03
Acrophase (ZT)	15.8 ± 0.1	16.0 ± 0.1

Data are mean (standard error range) or ± standard error of the mean.

[a] Pretreatment assessments were performed for 10 days without dosing, of which days 3–10 were used for analysis.

*$p < 0.05$ versus SAMR1 mice.

SAMP8, senescence-accelerated mouse prone-8; SAMR1, senescence-accelerated mouse resistant-1; ZT, Zeitgeber time.

Statistical analyses were performed using linear mixed-model analysis (with strain and time-bin as fixed effects and animal as a random effect), followed by Fisher's least significance difference test for log-transformed running wheel activity data.

Supplementary Figure 1. Effect of lemborexant (LEM) on total vigilance states over 23.5 h. In a randomized crossover design, mice (n = 8 per strain, per treatment) received single oral doses of vehicle, LEM 3 mg/kg, and LEM 30 mg/kg at Zeitgeber time 0:00–0:30. Data are mean ± standard error of the mean. *$p < 0.05$ versus senescence-accelerated mouse resistant-1 (SAMR1) vehicle. Statistical analyses were performed using linear mixed-model analysis (with treatment, strain, and their interaction as fixed effects and animal as a random effect), followed Fisher's LSD test (SAMR1-vehicle versus SAMP8-vehicle) or Holm's adjustment (versus vehicle) in each strain. REM, rapid eye movement; SAMP8, senescence-accelerated mouse prone-8.

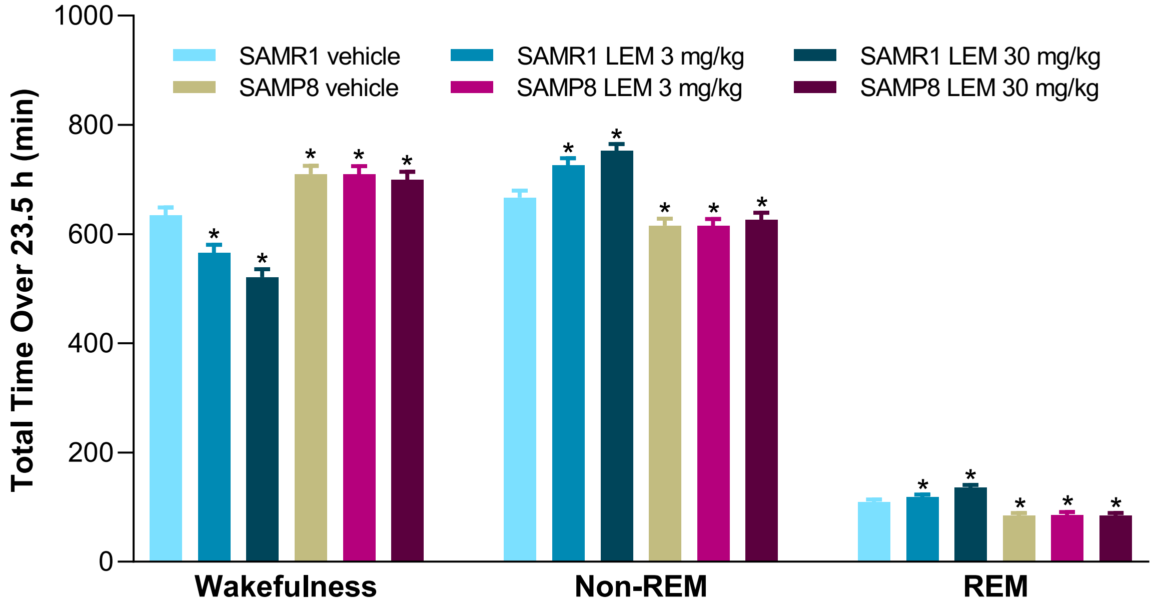

Supplementary Figure 2. Effect of lemborexant (LEM) on the wakefulness diurnal ratio. In a randomized crossover design, mice (*n* = 8 per strain, per treatment) received single oral (po) doses of vehicle, LEM 3 mg/kg, and LEM 30 mg/kg at Zeitgeber time 0:00–0:30. Data are mean ± standard error of the mean. $^{*}p < 0.05$ versus senescence-accelerated mouse resistant-1 (SAMR1) vehicle. $^{\dagger}p < 0.05$ versus senescence-accelerated mouse prone-8 (SAMP8) vehicle. Statistical analyses were performed using linear mixed-model analysis (with treatment and strain, and their interaction as fixed effects and animal as a random effect), followed Fisher's LSD test (SAMR1-vehicle versus SAMP8-vehicle) or Holm's adjustment (versus vehicle) in each strain. REM, rapid eye movement.

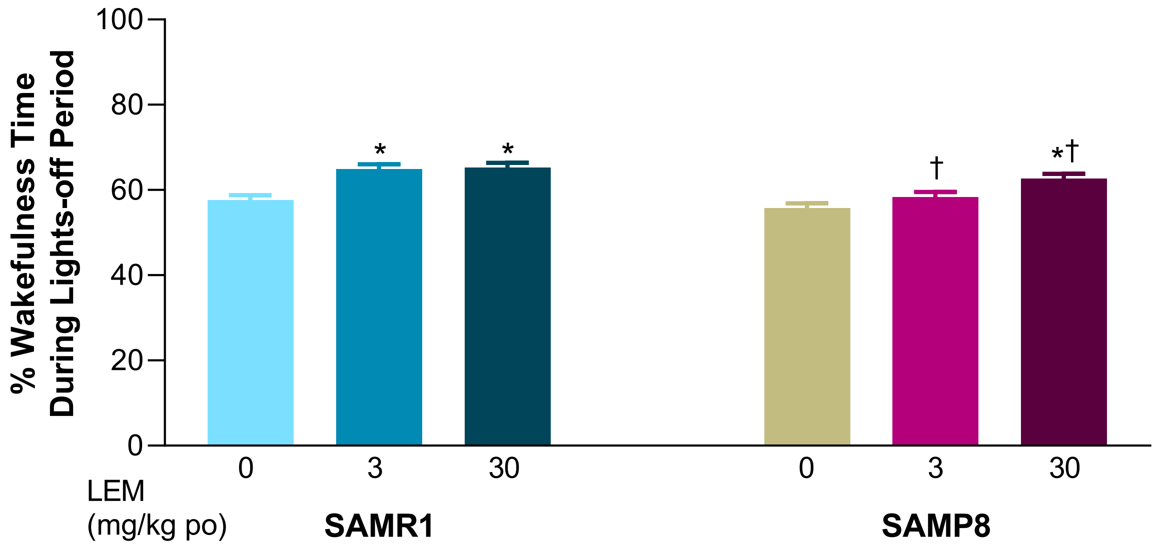

Supplementary Figure 3. Effect of lemborexant (LEM) on A, C) vigilance bout counts and B, D) bout duration during the lights-on and lights-off periods. In a randomized crossover design, mice (n = 8 per strain, per treatment) received single oral doses of vehicle, LEM 3 mg/kg, and LEM 30 mg/kg at Zeitgeber time 0:00–0:30. Data are mean ± standard error of the mean. *$p < 0.05$ versus senescence-accelerated mouse resistant-1 (SAMR1) vehicle. †$p < 0.05$ versus senescence-accelerated mouse prone-8 (SAMP8) vehicle. Statistical analyses were performed using linear mixed-model analysis (with treatment as fixed effects and animal as a random effect), followed by Fisher's LSD test (SAMR1-vehicle versus SAMP8-vehicle) or Holm's adjustment (versus vehicle) in each strain. REM, rapid eye movement.

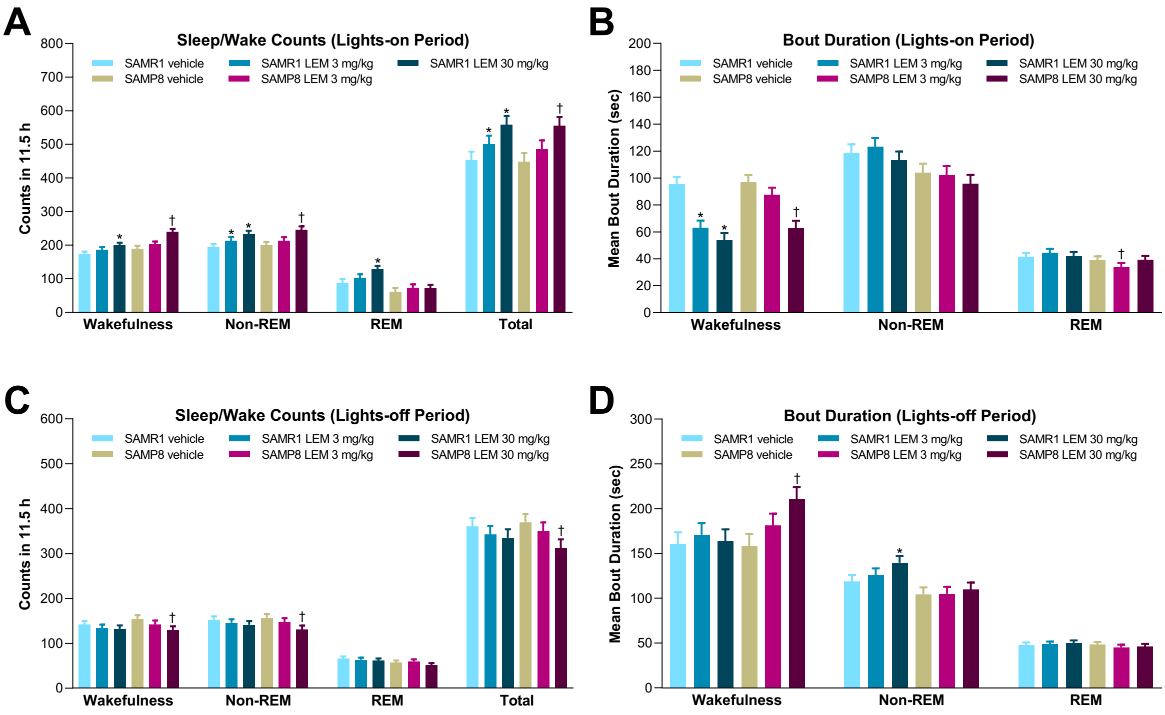

Supplementary Figure 4. Diurnal running wheel activity in vehicle- and lemborexant (LEM) 30 mg/kg–treated A–F) senescence-accelerated mouse resistant-1 (SAMR1) mice ($n = 12$) and G–L) senescence-accelerated mouse prone-8 (SAMP8) mice ($n = 11$–12) during the pretreatment, treatment, and posttreatment phases of the study. Pretreatment assessments were performed for 10 days without dosing, of which days 3–10 were used for analysis. Mice were then dosed orally with vehicle or LEM 30 mg/kg at the onset of light (Zeitgeber time [ZT] 0:00–0:30) each day for a further 10 days during the treatment phase. Posttreatment phase assessments (no dosing) were then performed for 10 days. The shaded area indicates the lights-off period. Red arrows indicate a significant within-strain difference ($p < 0.05$) between LEM and vehicle during 6-h bins (ZT 0–6, 6–12, and 18–24).

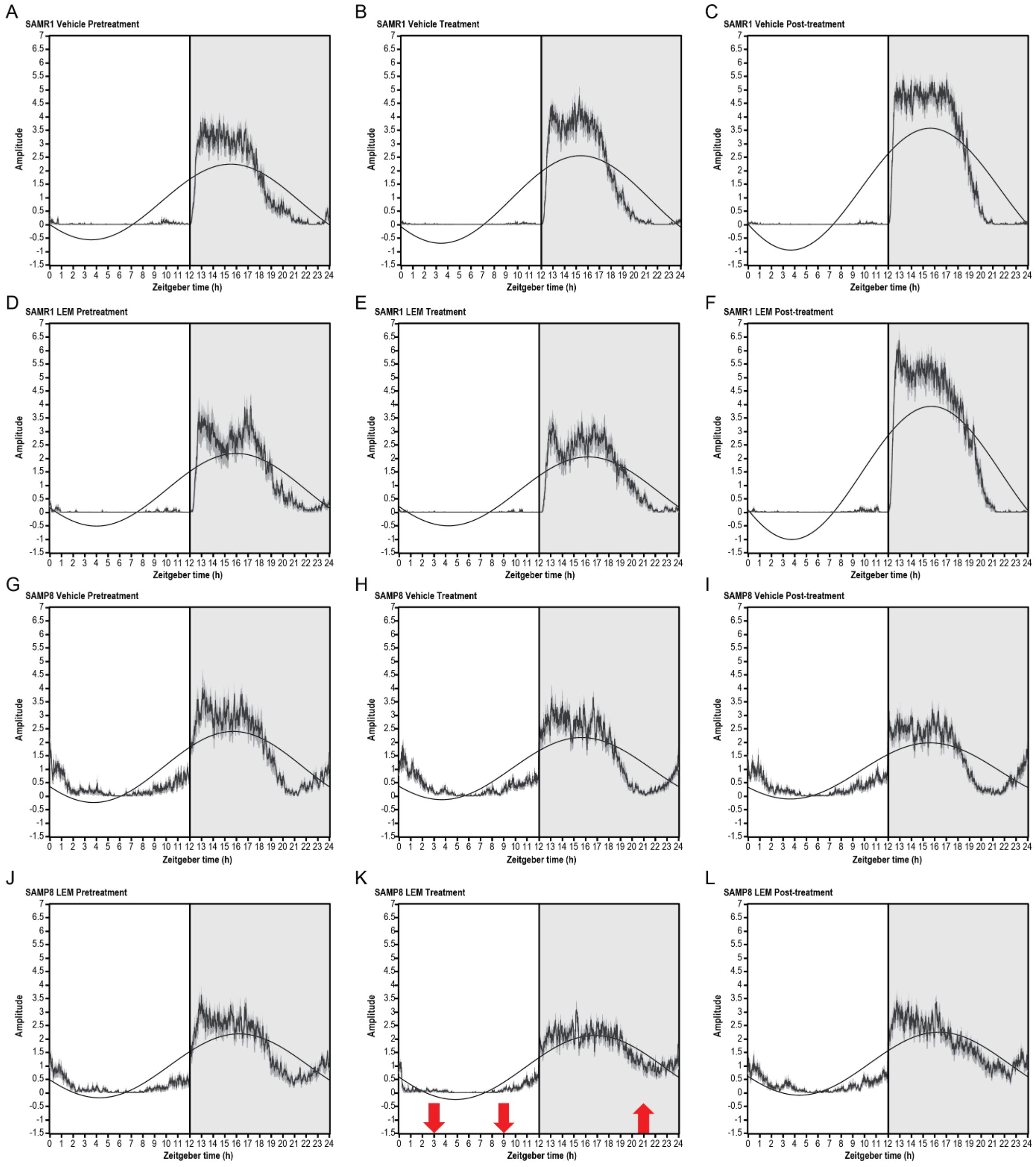

Supplementary Figure 5. Diurnal running wheel activity in senescence-accelerated mouse prone-8 (SAMP8) mice (n = 23) and senescence-accelerated mouse resistant-1 (SAMR1) mice (n = 24) during the pretreatment phase. Pretreatment assessments were performed for 10 days without dosing, of which days 3–10 were used for analysis. The shaded area indicates the lights-off period. Red arrows indicate a significant difference ($p < 0.05$) between SAMP8 and SAMR1 mice.

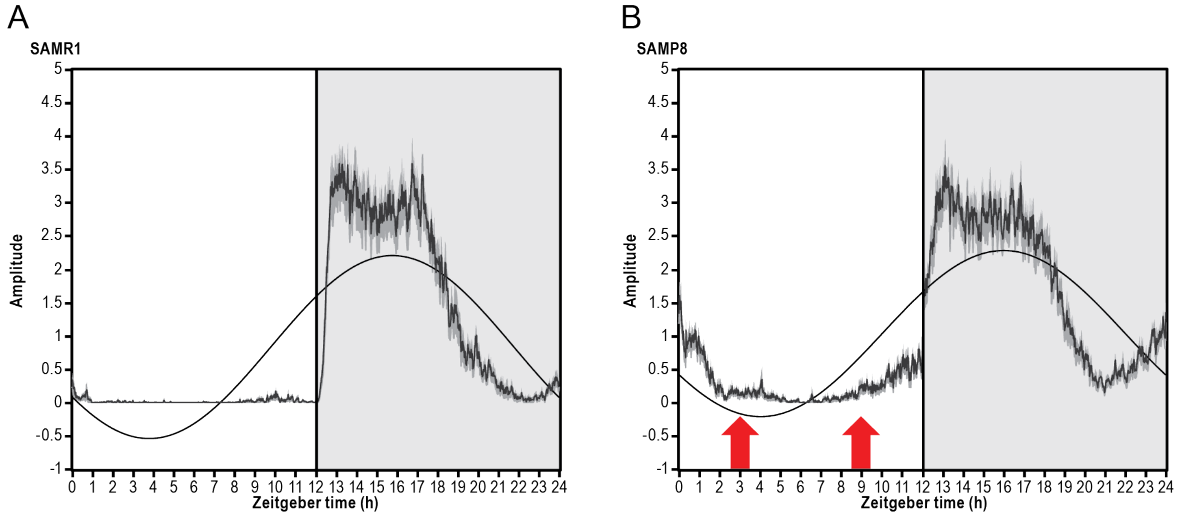

Supplement: Supplementary Material [file jad-81-jad201054-s001.pdf]
